# Histopathological Coexistence of Extragenital Lichen Sclerosus and Morphea in a Single Lesion

**DOI:** 10.7759/cureus.12215

**Published:** 2020-12-22

**Authors:** Reema R Almuqati, Jehad Hariri, Mohammed Abduljabbar

**Affiliations:** 1 Dermatology, Dr. Soliman Fakeeh Hospital, Jeddah, SAU; 2 Dermatology, King Abdulaziz University Hospital, Jeddah, SAU

**Keywords:** atrophy, coexistence, lichen sclerosus, morphea

## Abstract

Lichen sclerosus (LS) and morphea are two infrequent inflammatory dermatoses of unknown etiology. LS is characterized by, polygonal, bluish-white, slightly elevated papules that coalesce into plaques, which become increasingly atrophic overtime.it mostly affects genitals, however, it can affect any site on the skin and mucosa. Morphea characterized by, erythematous to violaceous patches or plaque with a white, sclerotic center, and the outer edge of the lesions take on the characteristic violaceous ring. The overlapping clinical and histopathologic features of both LS and morphea in the same patient have led some to speculate that they may have a common pathologic link or that both conditions represent the same disease spectrum. The coexistence of LS and morphea in the same lesion is a rare finding. We present a patient, who was diagnosed with what appeared clinically to be extragenital LS, but with histopathologic features of both LS and morphea.

## Introduction

Lichen sclerosus (LS) (also known as lichen sclerosus et atrophicus) is an uncommon, chronic, progressive inflammatory dermatosis of the skin and mucosa. It affects the epidermis and superficial dermis. This mucocutaneous disease has a high predilection for the anogenital area. Extragenital presentations of lichen sclerosus are common in involving the skin and are extremely rare in involving the oral mucosa [1]. The first description of lichen sclerosus was in 1887 by Hallopeau as a form of atrophic lichen planus [2]. In 1940, the first scientists who distinguished lichen sclerosus et atrophicus as an independent disease were Montgomery and Hill [3]. Nowadays, the term lichen sclerosus is the preferred term since not all cases of LS exhibit atrophy as a histopathological feature, as there is usually an area of thickening and hyperplasia.

The prevalence of lichen sclerosus is difficult to assess and is probably underestimated. Some patients are asymptomatic, especially those presenting extragenital forms. The disease occurs in all ages and both sexes. It is more common in women than men, the male-to-female ratio varies between 1:3 and 1:10. There is a typical peak incidence in prepubertal children, and in postmenopausal women, and in men in their fourth decade [4]. The disease is more frequent in Caucasians [5].

The extragenital LS most commonly presents on the neck, shoulders, trunk, proximal extremities, flexor surfaces of wrists, and sites of trauma or pressure. Patients have various complaints, which vary from pruritus to some other forms of cutaneous irritation or even could be asymptomatic. Lesions begin as polygonal, bluish-white, slightly elevated papules. These papules coalesce into plaques, which with time become increasingly atrophic, and become wrinkled. Telangiectasia and follicular plugging may become prominent. The fragility of the flattened epidermal-dermal interface may give rise to bullous and hemorrhagic lesions [6]. 

On the other hand, morphea (also known as localized scleroderma) is an uncommon fibrotic disorder that affects primarily the dermis and may extend to underlying tissues. Early morphea lesions begin as erythematous to violaceous patches or plaques. Over time, the center becomes white, sclerotic, and the borders of the lesions take on the characteristic violaceous ring, then the lesions become hairless and anhidrotic with variable degrees of dyspigmentation [7].

The etiology of both LS and morphea remains unknown. However, there is evidence suggesting several causative factors for both diseases. LS was postulated to be predisposed to autoimmunity, genetic susceptibility, trauma, chronic irritation, hormonal alterations, and infectious factors [8]. 

Morphea has also been hypothesized to be related to infection, autoimmune disease, genetic susceptibility, trauma, and radiation [9]. From the clinical point of view, both diseases have demonstrated a high prevalence of other coexisting autoimmune diseases, such as autoimmune thyroiditis, vitiligo, alopecia areata, and diabetes mellitus [8,10-12].

Although morphea and LS have been separated both clinically and histologically, there are very few cases in the literature reporting coexistence of both diseases’ features in the same lesion, proposing an etiological link between them [13-15]. Here, we present a case study of a patient, who was diagnosed with what appeared clinically to be extragenital LS, but with histopathologic features of both LS and morphea.

## Case presentation

A 43-year-old Saudi female was presented to the clinic, complaining of itching on her upper back for about one year. She denied any discomfort elsewhere. She had a history of diabetes and hypothyroidism but denied any history of other dermatologic conditions. There was no family history of similar skin diseases.

**Figure 1:**
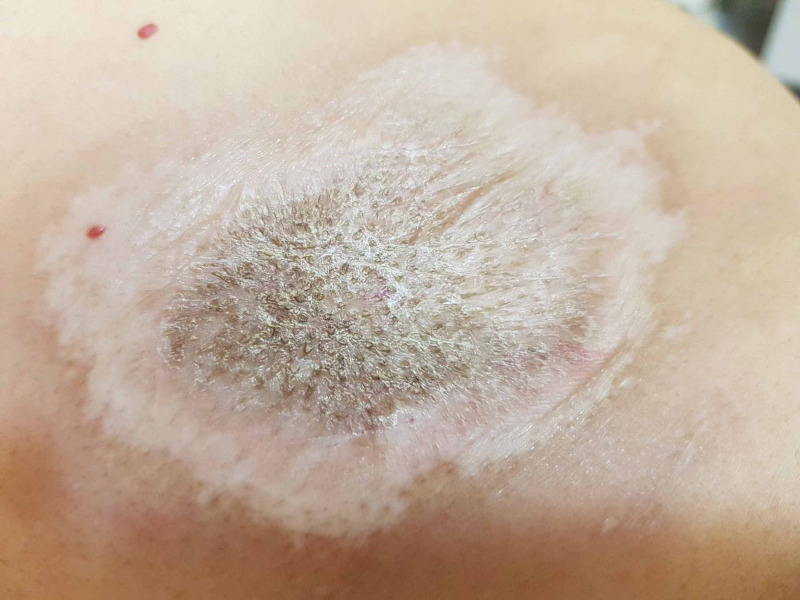
Hypopigmented atrophic plaque with wrinkled texture and follicular plugging.

On clinical examination, a hypopigmented atrophic plaque with wrinkled texture and follicular plugging involving the upper back was observed, measuring 7 cm by 10 cm (Figure 1).

**Figure 2:**
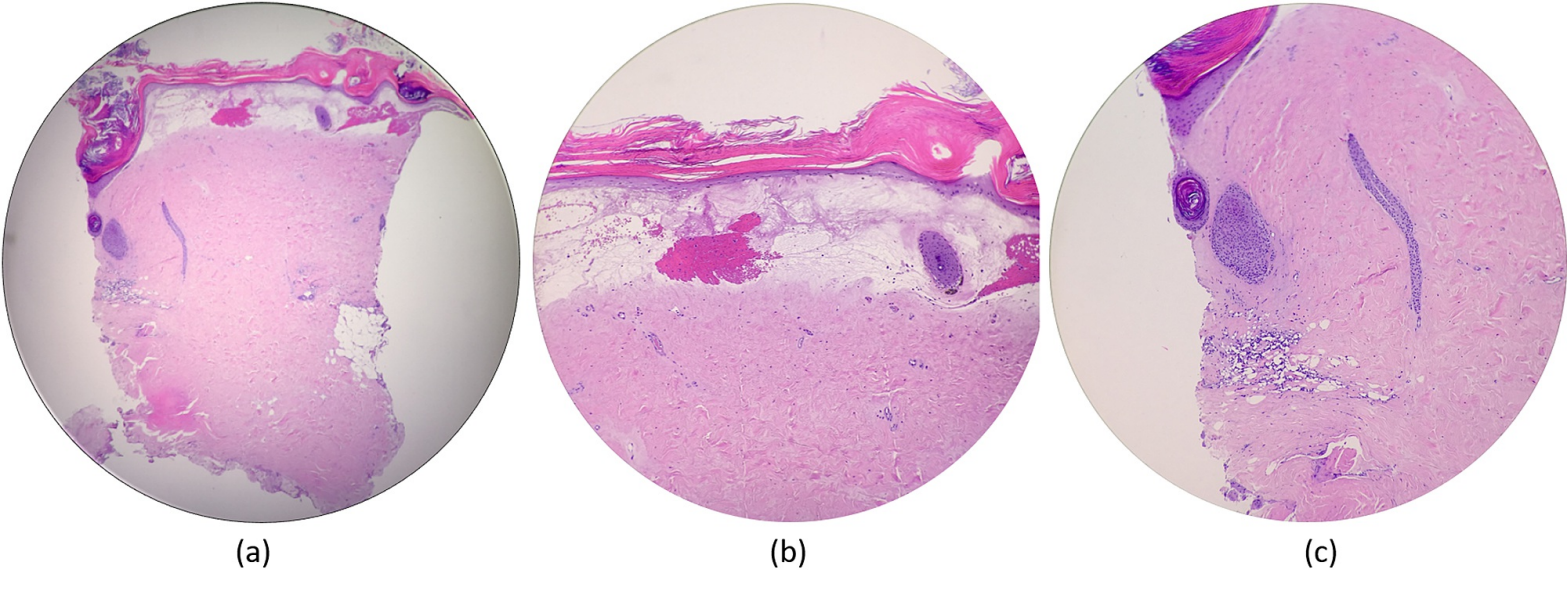
(a) Histologic sections demonstrate a squared-off biopsy specimen. (b) The epidermis shows hyperkeratosis, epidermal atrophy, and vacuolar interface damage. There is a homogenization of the papillary dermis, along with hemorrhage. (c) Follicular plugging is noted, as well as entrapped adnexa and a perivascular lymphoplasmacytic infiltrate.

The genital region was unaffected. A 4-mm punch biopsy was taken for histopathologic evaluation. The histopathologic examination shows mixed changes of both LS and morphea in the same lesion. Histologic sections demonstrate a squared-off biopsy with an epidermis showing hyperkeratosis, follicular plugging, epidermal atrophy, and vacuolar interface damage. There is a homogenization of the papillary dermis, along with hemorrhage. The deep dermis shows entrapped adnexa and a perivascular lymphoplasmacytic cell infiltrate (Figure 2).

The clinical and histopathological findings were consistent with extragenital lichen sclerosus along with morphea in the same lesion.

**Figure 3:**
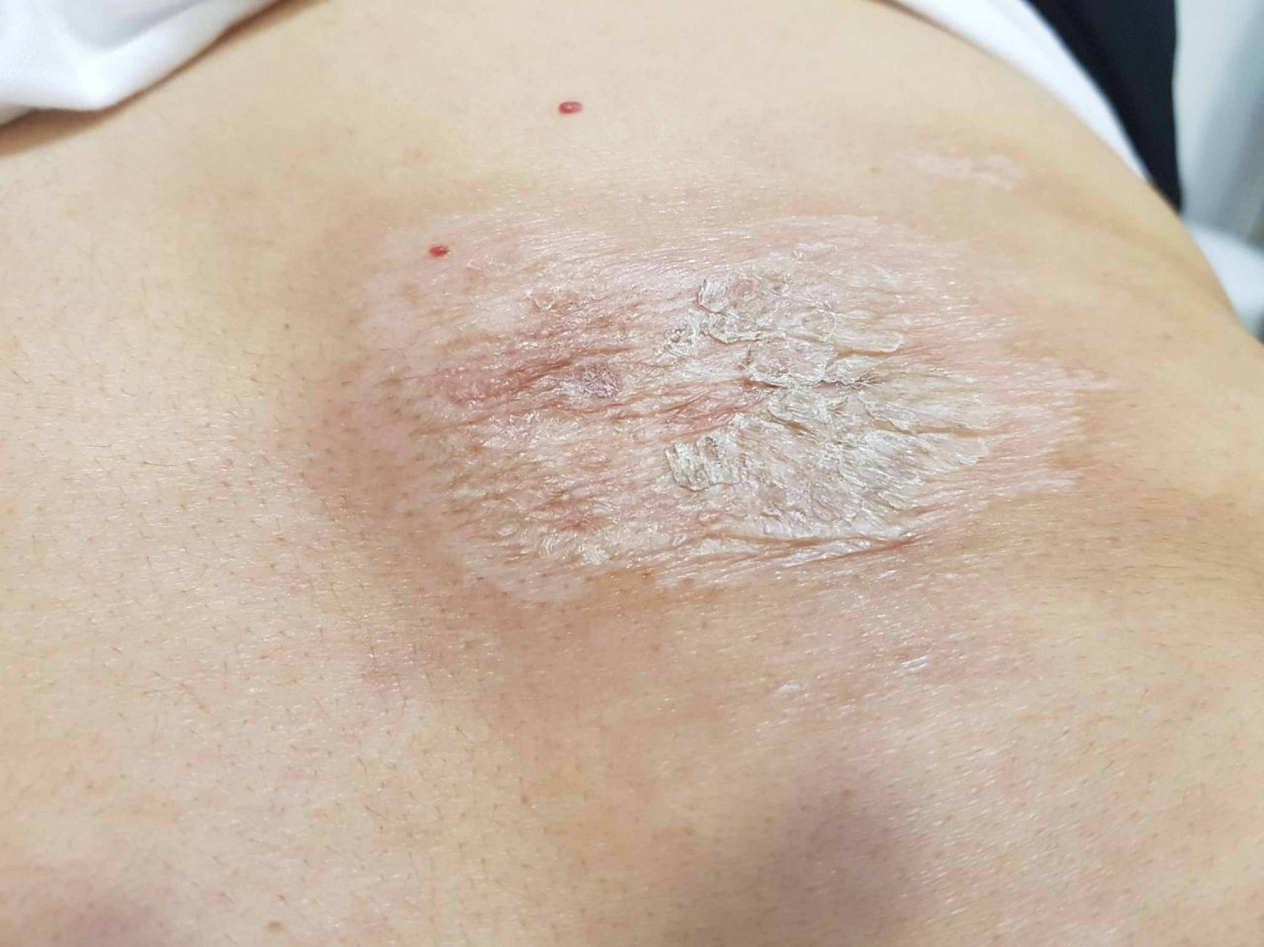
An improvement after 1 month of applying high-potency topical corticosteroid.

The patient was managed with high-potency topical corticosteroid ointment, twice daily for one month. The patient showed improvement (Figure 3).

After one month, the dosage was adjusted to be once daily for further two months.

## Discussion

Both LS and morphea are infrequent inflammatory dermatoses of unknown etiology. Extragenital lichen sclerosus and morphea may both present with white atrophic plaques which can be a diagnostic challenge for clinicians. The first description of the coexistence of these two diseases was in 1980 in 10 patients by Uitto et al; in seven of them, the coexistence of both diseases was observed in the same lesion [13]. The relationship between the two diseases has not yet been adequately explained and is still controversial. The reported cases in the literature of patients with coexisting histopathologic features of both conditions have led some to speculate that they may have a common pathologic link. Some other physicians speculate that both conditions represent the same disease. Peterson et al. proposed a categorization of LS as a subtype of morphea owing to the histologic similarities between the two diseases [16]. Some have referred to LS as subepidermal morphea [17]. On the contrary, other investigators argued that, and believe that, there are sufficient clinical and histologic differences between morphea and LS, and those coexistent lesions are coincidental.

Histopathologically, LS is characterized by follicular plugging, hyperkeratosis, epidermal atrophy, basal cell hydropic degeneration, and homogenized papillary dermis. On the other hand, morphea is characterized by the densely sclerotic dermis, squared-off biopsy, swollen collagen bundles, trapped adnexa, and perivascular lymphocytes and plasma cells.

A prospective multicentre study of 76 patients with morphea revealed an unexpectedly high percentage (38%) of genital LS and it was more frequent in patients with plaque morphea rather than other types of morphea. Lichen sclerosus could be the genital manifestation of morphea. Squamous cell carcinomas have been linked to genital LS with a lifetime risk of up to 5%. Thus, the examination of the genital region in morphea patients should be performed as a mandatory step to diagnose genital LS, and eventually to prevent or at least provide early diagnosis of genital carcinomas that might arise on them [18].

The mainstay of the therapy of LS is ultra-potent topical corticosteroids. It is very effective and the improvement is usually noticed in 75-90% of LS patients. It is the first-line treatment in LS regardless of age or gender. It is recommended to initiate the treatment with potent or ultra-potent topical corticosteroids for three months. Thereafter, usage continues once or twice weekly, to prevent disease flares.

The second line of treatment options is calcineurin inhibitors (tacrolimus and pimecrolimus). The effects are less in comparison to those of topical corticosteroids. Systemic treatment is occasionally indicated in refractory cases and includes methotrexate, retinoids, and cyclosporin. In widespread extragenital lichen sclerosus, narrow-band UVB phototherapy, and low-dose psoralen-UVA, have been proven to be effective [19]. The treatment options of limited morphea include topical class I or intralesional corticosteroids, tacrolimus, calcipotriene, and calcipotriol in combination with betamethasone dipropionate, lesion limited phototherapy, and imiquimod [7]. In case of widespread involvement of morphea, the use of methotrexate combined with systemic corticosteroids and UVA1 has the most convincing data supporting their use (Table 1) [20].

**Table 1:** The treatment options for LS and morphea. LS: lichen sclerosus

Disease Severity	Lichen Sclerosus	Limited Morphea
Mild to moderate	First-line: ultra-potent topical corticosteroids for three months. Thereafter, maintenance once or twice a week. Second line: topical calcineurin inhibitors (tacrolimus and pimecrolimus).	First-line: topical class I or intralesional corticosteroids, topical tacrolimus. Second line: lesion limited phototherapy, topical imiquimod, calcipotriene, and calcipotriol in combination with betamethasone dipropionate.
Sever or refractory	Systemic treatment includes methotrexate, retinoids, and cyclosporin. Widespread extragenital LS: narrow-band UVB phototherapy and low-dose psoralen-UVA.	Widespread morphea: methotrexate combined with systemic corticosteroids and UVA1.

## Conclusions

Lichen sclerosus and morphea are represented separately in clinical and histological manifestations. However, The coexistence of both diseases in the same lesion has been reported in a few cases in the literature which assume a possible etiological link between them. 
